# Morphological Correlates of a Combat Performance Trait in the Forked Fungus Beetle, *Bolitotherus cornutus*

**DOI:** 10.1371/journal.pone.0042738

**Published:** 2012-08-15

**Authors:** Kyle M. Benowitz, Edmund D. Brodie, Vincent A. Formica

**Affiliations:** 1 Mountain Lake Biological Station, Department of Biology, University of Virginia, Charlottesville, Virginia, United States of America; 2 Department of Biology, Swarthmore College, Swarthmore, Pennsylvania, United States of America; University of California Santa Barbara, United States of America

## Abstract

Combat traits are thought to have arisen due to intense male-male competition for access to females. While large and elaborate weapons used in attacking other males have often been the focus of sexual selection studies, defensive traits (both morphological and performance) have received less attention. However, if defensive traits help males restrict access to females, their role in the process of sexual selection could also be important. Here we examine the morphological correlates of grip strength, a defensive combat trait involved in mate guarding, in the tenebrionid beetle *Bolitotherus cornutus*. We found that grip strength was repeatable and differed between the sexes. However, these differences in performance were largely explained by body size and a non-additive interaction between size and leg length that differed between males and females. Our results suggest that leg size and body size interact as part of an integrated suite of defensive combat traits.

## Introduction

Male combat is an important component of mating systems in many animal taxa [1], [2]. Success in combat allows males to access and monopolize mates and the morphological traits involved in combat often experience strong sexual selection [3], [4], [5], [6]. Combat traits used in defense against aggressive attacks that also limit access to mates should be equally as important as corresponding aggressive traits, and yet defensive traits have received far less attention in the literature (but see [7], [8]). The morphological structures that have arisen through intrasexual selection and male-male combat (known collectively as combat traits) are some of the most diverse traits in the species that possess them (reviewed in [2]). While sexual dimorphism can arise from many forms of differential selection between the sexes [9], dimorphism in a putative combat trait can suggest that the trait has experienced strong sexual selection [1], [4], [10].

Arnold [11] suggested that one way to elucidate the process of selection on morphological traits is to measure performance of individuals in a relevant context. Then, by regressing the performance of the individuals on their phenotypic traits we can begin to draw a statistical and functional connection between traits and the contexts in which selection can act [12], [13]. Assessing the relationship between combat performance and morphology can be difficult under natural conditions, as aggressive encounters involve the actions of multiple individuals, are often unpredictable, and are difficult to observe. Therefore, most assessments of performance are made using controlled, manipulative experiments.

The relationship between morphology and combat performance has been examined in several taxa. In collared lizards (*Crotaphytus collaris*), bite force is correlated with head width, and also positively predicts the number of offspring produced in a breeding season, suggesting sexual selection on head morphology [14]. In tuataras (*Sphenodon punctatus*), the scaling relationship between body size and bite force differs between males and females, suggesting the presence of sexual selection on biting ability [15]. One of the few measured defensive performance traits used in a combat context is a dung beetle's ability to physically hold its position in a tunnel against an opposing force. Lailvaux et al. [7] found that horn size predicts tunnel holding ability in *Euoniticellus intermedius*. This relationship suggests that larger-horned males should have greater success restricting access to females; however, it is not the horns themselves that allow males to grip the tunnel, suggesting that there are other morphological traits that covary with horn size and provide larger males with the ability to defend their tunnels.

### Study System

**Figure 1:**
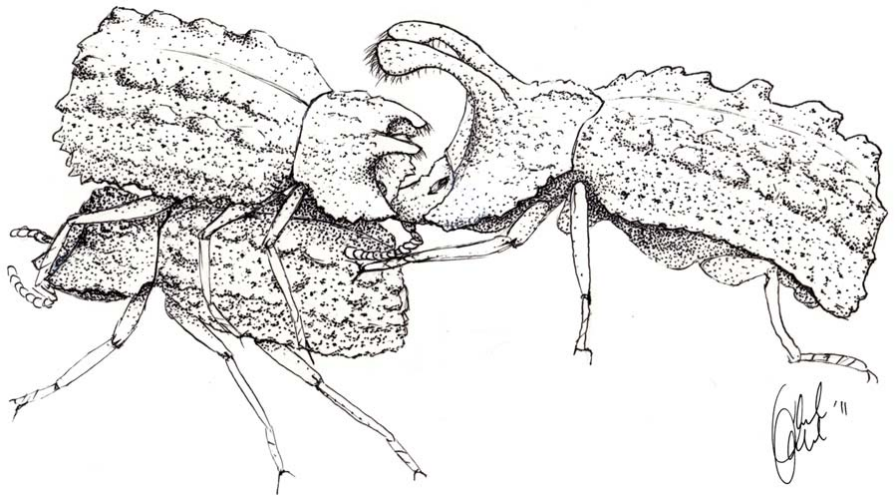
A small *B. cornutus* male exhibiting the use of the grip strength in the wild. The small male is courting the female while the large male attempts to use his clypeal horns to dislodge the courting male. While these images illustrate an attack from the front of the courting male, we have observed attacks from behind as well as perpendicularly to the defensive male (personal observation). Attacks also occur during mate guarding, when the male and female are oriented in the same direction. Illustration by Ariel Kahrl, from the supplemental video.

*Bolitotherus cornutus* is a tenebrionid beetle that is sexually dimorphic with respect to the presence of horns. Males use their two sets of horns, clypeal and thoracic, to compete for mates. Unlike many species of scarab beetles that exhibit male dimorphism for horns with major and minor morphs [7], [16], [17], male *B. cornutus* possess a continuous range of horn and body sizes [3], [18]. *Bolitotherus cornutus* perform reproductive behaviors on the surface of polypore shelf fungus growing on dead wood [3], [19]. Mating pairs engage in a courtship ritual in which the male grips the female's elytra, with his thorax over the end of her abdomen ([20] Figure 1; Video S1). Courtship often lasts several hours, and is a necessary precursor to copulation. During a copulation attempt, the male reverses position on top of the female so that both individuals point the same direction and their abdomens are aligned. If the courtship is successful, the female opens her anal sternite and copulation takes place. Following copulation, the male remains on top, facing the same direction as the female, and mate-guards her. The male remains in this position for several hours, preventing other males from courting the female.

During both courtship and guarding, other males often attempt to dislodge the male by prying him off of the female with their thoracic and clypeal horns ([3] Figure 1; Video S1). The use of the attacking male's horns is varied; occasionally both sets of horns are used like forceps to grip the center of the defending male to pry him off the back of the female, but other times the attacker will use his clypeal horns and try to peel off the defensive male from either his front or rear. If attacked during the courting or guarding stage, the defending male hooks all six tarsi under the female to hold his position. If a male is displaced by another male during courtship, he usually leaves the bracket immediately and does not copulate with the female. Additionally, this species has high variability in fertilization precedence [21]; therefore, removal during guarding even after insemination might also result in a loss of fitness to the defeated male. The frequency of attacker success is unknown in this species, but data from a sample of recorded observations suggest that larger males are more likely to initiate and win fights (Formica unpublished data; N = 15).

Sexual selection in *B. cornutus* favors larger horns [22] and larger body size [18], especially in low density populations. Interactions among conspecifics also lead to strong social selection—males with social groups composed of smaller males obtained more copulations than those with social groups composed of larger males [18]. These results suggest that the behavioral mechanism that influences sexual selection on horns and body size is the ability of larger males to block access to females. However, previous studies [3], [18], [22] did not further investigate the potential functional relationships among traits that might underlie selection. The goal of the current study was to examine the morphological correlates of grip strength, a defensive trait used by *B. cornutus* during combat, as a first step to elucidating the behavioral mechanisms that play a role in the previously observed sexual and social selection. We hypothesized grip strength would be a highly repeatable trait and that grip strength would be sexually dimorphic in *B. cornutus.* We also hypothesized that grip strength would covary with body size (elytra length) and femur length.

## Materials and Methods

All *B. cornutus* were collected from populations of *Ganoderma applanatum* and *Fomes fomentarius* on and around Mountain Lake Biological Station (MLBS) property in Giles County, Virginia, in June 2011 with written permission from the Director of the Station. Collection of animals followed all guidelines required by MLBS; no endangered or threatened species were collected. Beetles were then housed in a temperature-controlled room at MLBS at 16°C with 18 hours of light per day, to simulate early summer weather conditions. Beetles were kept individually with a small piece of *Ganoderma tsugae* for food.

**Figure 2:**
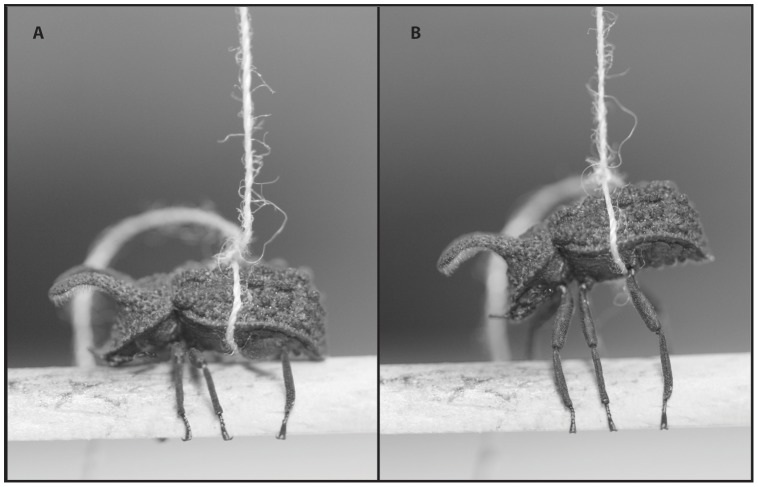
A large male *B. cornutus* undergoing a grip strength trial. (A) The male grips the dowel rod in a similar fashion to how males grip females during courtship and guarding in the wild. (B) The same male at maximum resistance just before releasing the dowel rod.

Forty-one females and forty-three males were assayed for grip strength in July 2011. During these assays, each beetle was tethered between the second and third set of legs with a piece of thread. The beetle was then induced to grip a 6.35 mm diameter dowel rod, which was cut in half longitudinally and suspended 12 mm above the device surface (Figure 2). Beetles were not tested until they wrapped at least five legs around the bottom edge of the rod. The thread extended from the beetle vertically, through two pulleys, and attached to a digital force gauge (Shimpo, FGV-XY-0.5, Tensitron) placed on a horizontal platform. The force gauge was moved away from the beetle on a platform (VelmexSenc150, Bloomfield, NY) at a constant speed, thereby pulling the beetle vertically until it released the dowel rod (Figure 2). The gauge recorded the force (in grams) required to displace the beetle from the dowel rod (hereafter “grip strength”). The value of force in grams is not exact due to friction acting on the pulleys and the mass of the beetles (mean mass = 0.11±0.005 g SE), but provides a measure of relative performance as all beetles were tested on the same apparatus. Each beetle was tested three times, with a period of 12–48 hours between trials to allow individuals to rest. Before each trial, every beetle was examined for missing tarsi and leg sections, to insure that all legs were in fact gripping the dowel in a similar manner. This method most closely simulates a prying attack where attacking males attempt to lift defending males vertically off the female; while this method does not simulate all of the different attacks that have been observed (e.g., being peeled off the female), it was the most repeatable way to assess relative abilities of beetles to grip a surface.

After the grip trials were completed, all beetles were imaged in high resolution on a flatbed scanner (Epson Perfection V600 Photo, Suwa, Nagano, Japan). Measurements of elytra length and pronotum width were taken for all individuals, as well as thoracic and clypeal horn length for males. We also removed one back leg from each individual to measure leg traits. In order to measure several morphological traits that might relate to a mechanism for differences in grip strength, we measured the length of several leg parts (tarsus, tibia, and femur). However, due to inconsistent landmarks, three-dimensional structures, and inconsistencies in the break point during removal of the leg, femur length provided the most reliable and repeatable measurements. We therefore used femur length as a proxy for overall leg size, assuming that femur length correlates positively with overall leg length, diameter and muscle mass. All measurements were made using ImageJ software.

All beetles with broken or injured legs were excluded from the analysis, leaving a measured sample of 31 males and 30 females for the final analysis. The repeatability of grip strength was obtained by calculating the intra-class correlation coefficient, *t*, using a general linear mixed model (GLMM) with the individual ID as a random effect. Subsequent analyses of grip strength used the average of three measurements for each individual. Correlations among grip strength and morphological variables were estimated using Pearson's coefficient. We conducted a t-test to test whether the sexes differed in grip strength. Because femur length, elytra length, and sex are highly correlated (Table 1), we performed a multivariate GLM with all interaction terms to simultaneously analyze the effects of these variables on grip strength.

**Table 1:** Correlations among morphological traits (mm) and grip strength (g) in the tested sample of *B. cornutus* (N = 61).

	Mean Grip Strength	Elytra Length	Femur Length	Pronotum Width	Thoracic Horn Length*	Females Mean ± SE	Males Mean ± SE
Elytra Length	0.66					6.68±0.58	6.91±0.44
Femur Length	0.71	0.76				2.82±0.25	3.24±0.19
Pronotum Width	0.68	0.95	0.75			3.80±0.33	3.92±0.31
Thoracic Horn Length*	0.61	0.87	0.78	0.92			2.55±1.06
Clypeal Horn Length*	0.56	0.67	0.66	0.68	0.71		0.61±0.21
Mean Grip Strength						53.34±15.51	71.47±16.92

All correlations are significant (P<0.001). *Females do not possess horn structures in this species; therefore, correlations between horns and other variables are only calculated for males (N = 31).

## Results

The repeatability of grip strength among all individuals across the three trials was 0.58. The repeatability for males (*t* = 0.43) was slightly lower than for females (*t* = 0.62), but still relatively high compared to other behavioral traits [23]. Grip strength was significantly greater in males than in females (t_59_ = 4.36, P<0.001).

**Figure 3:**
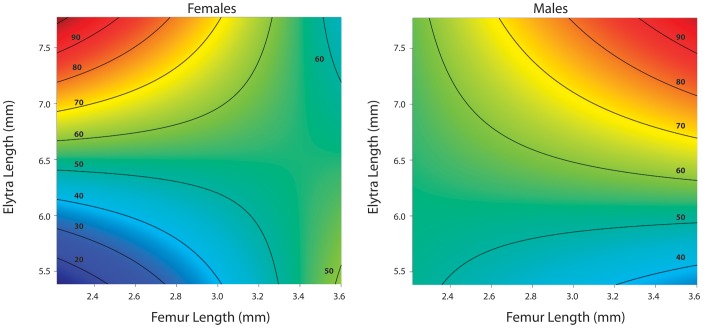
Response surface showing the multivariate relationship of grip strength and morphology for each sex (based on the fully-justified GLM model). Males and females differ in the overall relationship among grip strength, body size, and femur length. Warmer colors (red) signify areas of higher grip strength, while cooler colors (blue) signify areas of weaker grip strength.

When morphological variation and sex were simultaneously considered, simple sex differences in grip strength were not evident. Elytra length positively covaried with grip strength overall. Sex, femur length, and elytra, interacted to influence grip strength (Table 2; Figure 3); no other parameters were significant. Inspection of the response surface predicting grip strength in each sex shows that males with longer femurs and bigger body size exhibited the strongest grip, whereas females with relatively shorter femurs and bigger body size had the greatest grip strength.

**Table 2:** Parameter estimates and significance values for fully justified GLM predicting grip strength (N = 61). Bold variables were significant (α = 0.05).

	Estimate (b)	Std. Error	*t*-ratio	P value
**Elytra Length**	**14.31**	**6.00**	**0.12**	**0.02**
Femur Length	1.75	14.40	2.39	0.90
Sex	−3.97	2.78	−0.68	0.16
Elytra × Femur	−7.36	10.90	−0.14	0.50
Elytra × Sex	−2.29	5.98	−0.75	0.70
Femur × Sex	−10.85	14.40	−0.38	0.45
**Sex × Femur × Elytra**	**−24.63**	**10.88**	**−2.26**	**0.03**

## Discussion

An individual beetle's grip strength proved to be a highly repeatable performance trait under controlled conditions. This individual consistency and variance among individuals are critical requisites for selection to act on any performance or behavioral trait [24], [25], [26]. Though most studies of combat traits have focused on offensive weaponry, a male's ability to repel an attack should be an equally important determinant of a fight's outcome. Eberhard et al. [8] provide one example of a defensive structure in the measurement of sheath size in the weevil *Parisoschoenus expositus*. Another example is tunnel defense performance described in various dung beetle species [7], [27]. Neither study, however, integrates analysis of a specific morphological structure that could mechanistically affect the outcome of defense during combat.

The apparent sexual dimorphism in grip strength in *B. cornutus* seems to result from a complex relationship between body size and leg length that differs between the sexes. Visualization of the grip strength response surfaces for the two sexes (Figure 3) shows that both elytra and femur contribute positively to grip strength in males. In females, on the other hand, grip strength actually decreases with increasing femur length at large elytra lengths. Thus, performance scales differently with multivariate morphology in the two sexes. These sex differences are the first lines of evidence that grip strength may be under sexual selection. We do not suggest that femur length or elytra length are the functionally important morphological variables that affect gripping ability. Rather, our data suggest that femur length and elytra could be part a suite of traits (possibly including traits such as leg muscle mass, tarsus length, tarsal morphology, and even behaviors) that influence differences in grip performance and therefore might experience indirect sexual selection. Leg and body size could experience selection through their use in attack and courtship, or indirectly through correlations with other traits like horn length. In order to test these alternatives, fitness needs to be measured in the wild and a selection study needs to be conducted, where a path model is used to elucidate whether grip strength mediates the relationship between morphology and fitness [11], [28].

## Supporting Information

Video S1 **Combat between two forked fungus beetle (** ***Bolithoterus cornutus*** **) males.** The larger attacking male uses his clypeal horns in an attempt to dislodge the smaller male. The smaller male was previously courting the female and during the combat grips her with all six legs. The video has been sped up to 5x actual speed.
(AVI)
